# Modelling the landscape of palliative care for people with dementia: a European mixed methods study

**DOI:** 10.1186/1472-684X-12-30

**Published:** 2013-08-12

**Authors:** Steve Iliffe, Nathan Davies, Myrra Vernooij-Dassen, Jasper van Riet Paap, Ragni Sommerbakk, Elena Mariani, Birgit Jaspers, Lukas Radbruch, Jill Manthorpe, Laura Maio, Dagny Haugen, Yvonne Engels

**Affiliations:** 1 Research Department of Primary Care & Population Health, University College London, London, England; 2 Scientific Institute for Quality of Healthcare (IQ healthcare), Radboud University Nijmegen Medical Centre, Nijmegen, The Netherlands; 3 Palliative Care Research Centre, Department of Cancer Research and Molecular Medicine, Faculty of Medicine, Norwegian University of Science and Technology, Trondheim, Norway; 4 Department of Psychology, University of Bologna, Bologna, Italy; 5 Department of Palliative Medicine, University Hospital Bonn, Bonn, Germany; 6 Centre for Palliative Medicine, Malteser Hospital Bonn/Rhein-Sieg, Bonn, Germany; 7 Social Care Workforce Research Unit, Kings College London, London, England; 8 European Palliative Care Research Centre, Department of Cancer Research and Molecular Medicine, Faculty of Medicine, Norwegian University of Science and Technology, Trondheim, Norway; 9 Department of anesthesiology, Pain and Palliative Medicine, Radboud University Nijmegen Medical Centre, Nijmegen, The Netherlands

## Abstract

**Background:**

Palliative care for people with dementia is often sub-optimal. This is partly because of the challenging nature of dementia itself, and partly because of system failings that are particularly salient in primary care and community services. There is a need to systematize palliative care for people with dementia, to clarify where changes in practice could be made.

To develop a model of palliative care for people with dementia that captures commonalities and differences across Europe, a technology development approach was adopted, using mixed methods including 1) critical synthesis of the research literature and policy documents, 2) interviews with national experts in policy, service organisation, service delivery, patient and carer interests, and research in palliative care, and 3) nominal groups of researchers tasked with synthesising data and modelling palliative care.

**Discussion:**

A generic model of palliative care, into which quality indicators can be embedded. The proposed model includes features deemed important for the systematisation of palliative care for people with dementia. These are: the division of labour amongst practitioners of different disciplines; the structure and function of care planning; the management of rising risk and increasing complexity; boundaries between disease-modifying treatment and palliative care and between palliative and end-of-life care; and the process of bereavement.

**Summary:**

The co-design approach to developing a generic model of palliative care for people with dementia has placed the person needing palliative care within a landscape of services and professional disciplines. This model will be explored further in the intervention phase of the IMPACT project.

## Background

Improving palliative care for people with dementia is a policy objective Europe-wide [1]. Palliative care services have developed within national health care systems, resulting in both diversity and inequity of provision [2], and lack of unified standards and accepted definitions [3]. This has prompted the search for a common language for palliative care [4] which clarifies distinctions between palliative and end-of-life care, and which would allow general requirements for palliative care services to be described, together with specific requirements for each service type [5]. From this perspective, proposed by the World Health organisation, the principles of palliative care should be applied as early as possible in the course of any chronic, ultimately fatal illness. Palliative care is not synonymous with end-of-life care, but subsumes it [6]. Palliative care for people with cancer is relatively well developed, in terms of its conceptual framework and evidence base [7]. The evidence base to guide practice with those dying with dementia is less well developed, although now evolving [8].

Providing palliative care for people with dementia faces special challenges. Dementia has a lengthy illness trajectory, in which there is a progressive deterioration [9,10] often punctuated by steeper declines caused by bouts of acute illness [11]. The overall estimated median survival time from onset of dementia to death is 4.1 years for men and 4.6 years for women, with longer survival times in those with early onset dementia (in their 60s) [12]. Although sometimes described as a long-term condition, there are no disease-modifying treatments for dementia, only symptom-modifying ones making it a condition requiring palliation.

The need for a palliative care approach to patients with dementia and their carers is not routinely grasped in primary care. For example, a study from Scotland (where general practitioners (GPs) are paid to maintain palliative care registers) found that only 20% of patients with dementia were on palliative care registers, and they were identified as needing palliative care only at the end of the disease process [13]. A similar study from UK primary care found that family members (not professionals) were the main care coordinators, and that transitions between services - for example from specialist care to general practice - created challenges for them [14]. Likewise, in a large Belgian study GP-patient conversations were less frequent among those with (45%) than those without (73%) dementia. The authors commented that a palliative care approach appeared to be initiated too infrequently [15]. Sub-optimal management of people dying with or from dementia occurs in other settings, too. A survey of geriatric and palliative medicine professional societies in Europe showed that palliative care in long-term care facilities and in geriatric wards was less developed than in specialist services, and that family and paid care workers are not well prepared to support older patients living at home or in nursing homes needing palliative care [16]. This difficulty in creating and maintaining the kind of holistic care that Warner and colleagues call “wrap around” support for people with dementia, is visible across Europe [17], as are the inequities in palliative care provision [2].

There is a need to systematize palliative care for people with dementia, to clarify where changes in practice could be made. There is also a need to evaluate attempts to change practice, acknowledging the contextual complexities of dementia care. Comparative studies of palliative care services in different countries can advance policy-making, but to do so they need to capture the complexities of provision and be grounded in social science models [18]. Such research also needs to consider how and whether services meet needs along the trajectory of the illness [19].

Two factors appear to maintain the unsatisfactory situation in which “wrap around” (holistic) support for people with dementia is uncommon in Europe. The first is the nature of dementia itself. The second is a set of system failings that are particularly salient in primary care and community services.

### The nature of dementia

Few people with dementia can express their preferences for palliative care at the time that they need it [20,21]. Professionals have to rely on a combination of information from advance care planning, if undertaken, their own knowledge and that of family carers, and their own clinical observations. Recognizing when a person with dementia is nearing the end of life is particularly challenging, and it is easy to see how the need to prepare for the end of life can so easily be missed [22,23]. Co-morbidities (particularly cardiovascular disease) complicate the clinical picture and may create a need for palliative care at any stage of the dementia process, since most people die with dementia rather than from it [24].

### System failings

The problem of variable quality of palliative care is particularly, although not exclusively, evident in community settings such as care homes (long term care facilities) and primary care services [25]. A pan-European study found a similar pattern across Europe [16]. Access to palliative care services and social support are two factors (alongside environmental and material resources) that determine whether older people with dementia can remain living in their own homes at the end of life [26].

General Practitioners (GPs) or family doctors in Europe may have clinical responsibility for patients needing palliative care (although the extent of this responsibility differs between countries) but the quality of their engagement is variable. A systematic review which included literature from the Netherlands, Belgium and the UK, among other countries, showed that barriers to GP-patient communication in palliative care include the unavailability of a GP, reluctance by both patients and families and their doctors to discuss a ‘bad’ prognosis, and failure by GPs to discuss former mistakes. Facilitators include GP availability, willingness to initiate discussion about a range of end of life issues, and anticipating scenarios of disease progression [27]. In the UK the use of the Gold Standard Framework (GSF) improves GP care processes, co-working and quality of palliative care, but its use is variable and the GSF’s direct impact on patients and families, especially where dementia is present, is not yet known [28].

Care home (residential and nursing home) residents usually have complex co-morbidities and many need palliative care. Reported problems in accessing palliative care for care home residents include: variable support from GPs, reluctance by GPs to prescribe appropriate medication, lack of support from other agencies, lack of out-of-hours support, costs of syringe drivers, limited expectations of and access to palliative care training, and poor pay and status for care home staff. Within the UK, care staff working with people with dementia are the least likely to have been able to access training [29] and turnover is high. Critical factors in improving end of life care for care home residents appear to be: developing clinical leadership, improving relationships with GPs, support from external advocates, and leverage of additional resources by adopting care pathways [30] but it is not known how these can be sustained.

### Systematising palliative care

Palliative care for people with dementia is less well systematized (in the sense of having structured care pathways) than that for people with cancer. The evidence base to guide practice in palliative and end of life care for older people with dementia is limited. There is a need to define good practice, and more needs to be known about the context of provision, about the effects of ageism and stigma, and about the influence of competing priorities and incentives [31]. In other words, we lack a generic model for palliative care in dementia, suitable for use in different health and care systems as a guide to service quality. Equally, we need to identify appropriate outcomes, so that good care can be characterized in terms of quality indicators and benchmarks and the effects of interventions can be measured.

### Evaluating innovative care

Intervention studies of new approaches to improve palliative care in dementia are needed, but are methodologically challenging. Evaluation of palliative care as a complex clinical and social intervention needs to acknowledge that it is influenced by a broader political, cultural and organisational context which is difficult to control. Case studies, participatory action research and before-and-after studies are useful ways of assessing the impact of contextual factors on palliative care services, when experimental studies like Randomised Controlled Trials are not feasible [32].

This paper is a contribution to the debate about palliative care for people with dementia. It reports on the first part of the EU-funded collaborative research project IMPACT (Implementation of quality indicators in Palliative Care sTudy), describing a technology development approach [33] used to construct a generic model of palliative care (Technology is used in its widest sense here, referring to any intervention). The IMPACT project follows the UK Medical Research Council’s (MRC) guidelines for the development and evaluation of complex interventions [34], beginning with modelling and pilot work which were carried out in work package 2 of the study. The generic model is designed to capture commonalities and differences in palliative care across Europe and is shaped as a landscape in which different quality indicators can be embedded. We discuss this landscape in terms of the insights it offers for the care of people with dementia. In this discussion we refer to palliative care as support and treatment given when disease-modifying therapy is no longer effective, or as in dementia, to a great extent not available.

### Modelling

The aim of IMPACT was to develop a model of palliative care for people with dementia that captures commonalities and differences across Europe. The model will be used to inform the targeting of interventions in the intervention phase of the IMPACT study.

The objectives were:

1) To scope the indexed, peer-reviewed scientific literature on palliative care, with an emphasis on services for people with dementia.

2) To scope the grey literature on policy and practice in palliative care, across five European sites (in England, Germany, Italy, Norway, & The Netherlands).

3) To construct organisational models of palliative care for each country using expert sources.

4) To synthesise findings from the literature with national organisation models to produce an over-arching model of palliative care for Europe which is patient centred.

**Figure 1:**
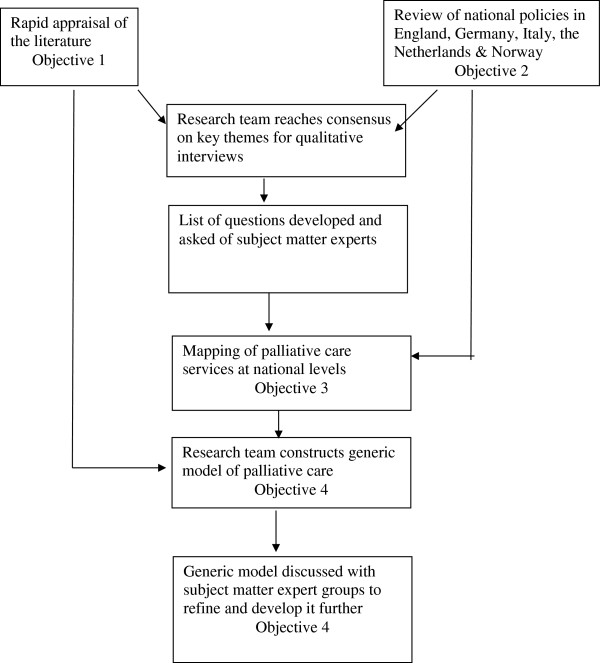
Developing a generic model of palliative care.

The methods used are summarised in Figure 1.

### Objective 1: review of research literature

Reviews of indexed peer-reviewed literature were undertaken, using rapid appraisal and critical interpretative synthesis methods [35]. A rapid appraisal approach was adopted to allow timely development of a usable model, and to inform the current implementation of policy. This approach is especially relevant when the different perspectives of the team members are essential for understanding the situation [36]. Influencing policy development and implementation is now a challenge for researchers in all countries with government-driven dementia strategies, and timing is important [37].

Critical interpretative synthesis, offers a more interpretive approach to literature reviews rather than systematically aggregating data. The purpose of critical interpretive synthesis is to construct theories grounded in the research. This approach was applied to reviews on barriers to good care, to generate practical methods to overcome these barriers and to change practice. Themes from this synthesis informed the design of a semi-structured interview schedule, together with themes from a review of the ‘grey literature’ (see below). Search strategies and findings of reviews on family carers’ perspectives of palliative care for people with dementia, and evaluations of professional education projects on palliative care for people with dementia, are reported elsewhere [38,39].

### Objective 2: narrative review

A narrative review of policy publications on and guidelines for palliative care practice (‘grey literature’) was carried out at each national research site. For example, the Netherlands research centre identified three relevant national guidelines, from the Nederlands Huisartsen Genootschap (Dutch College of General Practitioners), the Integraal Kankercentra Nederland (Dutch Association of Comprehensive Cancer Centres) and the Centraal Begeleidingsorgaan (Dutch Institute for Healthcare Improvement). Two legal documents were included because of their formative influence on palliative care services, the Algemene Wet Bijzondere Ziektekosten (Exceptional Medical Expenses Act) and the Wet Maatschappelijke Ondersteuning (Law of Social Assistance). In addition, they used an overview of the healthcare system in the Netherlands, including the complex payment system of health care professionals (e.g. general practitioners) [40] and a review of the organisation of palliative care in the Netherlands [41] to guide their summary of factors shaping palliative care.

The narrative reviews were used to inform the design of a semi-structured interview schedule for use with national experts The narrative reviews and the themes arising from the interviews (see Objective 3) were then used to create a national description of palliative care that took into account local variations, and this was then translated into English.

### Objective 3: constructing the model

A co-design approach [42] was used at each of the five national sites, with purposive sampling of national experts in policy, service organisation, service delivery, patient and carer interests, and research in palliative care, using a sampling framework designed for the purpose and supported by snow-balling methods [39]. The experiences of people with cancer or with dementia were represented by voluntary organisations advocating for people people with these conditions; that is, Alzheimer associations and cancer support organisations like Marie Curie Cancer Care. The sampling frame was a matrix of macro, meso and micro level organisations working with people with dementia in four settings: own home, care home, hospital, and hospice, as shown in Table 1. A semi-structured interview approach was chosen because this allowed the respondent to display their way of understanding their world, and to raise issues not anticipated by the interviewer [43].

**Table 1:** Sampling frame for recruitment of interview participants

**Domain**	**Primary care**		**Secondary care**	**Tertiary care**
**Setting**	**Own home**	**Care home (including care home with nursing)**	**Hospital**	**Hospice**
Direct providers of care - Practitioners & professionals* (micro level)				
Other available services** (meso level)				
National policy context*** (macro level)				

* Those clinical practitioners who provide dedicated palliative care within the settings, their management and their sources of funding.

** Other services available, including service management.

*** Those developing and implementing high level guidelines and policies designed to support high quality palliative.

The number of experts recruited at each site depended on reaching saturation, the point where no new themes were emerging from the interviews. The interview schedule developed from the interpretative synthesis and the ‘grey literature’ narrative reviews was adapted during the first interviews. Interviews were taped for transcription in the local language or captured using contemporaneous note-taking. Face-to-face interviews were preferred, but telephone interviewing was accepted when requested because it was more convenient for the informant. Interview transcripts and notes were analysed using a pragmatic thematic approach [44]. When no new codes emerged the research teams at each national site assumed that saturation had been reached. Five overarching themes were identified from the interviews as common factors which challenge the quality of palliative care across the five countries in the IMPACT study included in this study: Communication difficulties between services, and between professionals and patients and their families; the variable extent of structural/functional integration of services; the difficulties in funding of palliative care services; the problematic processes of care, including boundaries, definitions, knowledge, skills and inclusiveness; and time constraints in palliative care. These themes are presented in detail elsewhere [Davies N, Maio L, Van-Riet-Paap J, Mariani E, Jaspers B, Sommerbakke R, Grammatico D, Manthorpe J, Ahmedzai S, Vernoij-Dasen M, Engels Y: *Quality palliative care across Europe for cancer and dementia: International challenges*; 2013. Submitted].

### Objective 4: synthesis

A consensus meeting of the research team was organised to construct an over-arching European model or models of palliative care. In this meeting a modified nominal group technique was used to develop the generic model. Nominal groups are potentially powerful learning and development tools [45]. They have a particularly useful role in analysing health care problems [46], and can help bridge the gap between researchers and practitioners [47]. A nominal group approach designed for ill-structured problems was chosen, to allow for disagreements over problem definition, and to produce potential solutions that overlapped or varied widely in specificity. This requires the group to generate ideas, confirm that they are addressing the same problem, analyse the content of the ideas, categorise ideas and clarify the items in each category [48].

The approach to conceptual modeling followed the methods described by Kotiadis & Robinson [49]. A simplified model was abstracted from a detailed system description drawn up by subject matter experts in each national site, using the matrix described above and shown in Table 1. A second nominal group was conducted with members of the research team from all five sites, and also with a wider research advisory committee, to review, modify and validate the generic model.

### Findings & outputs

**Figure 2:**
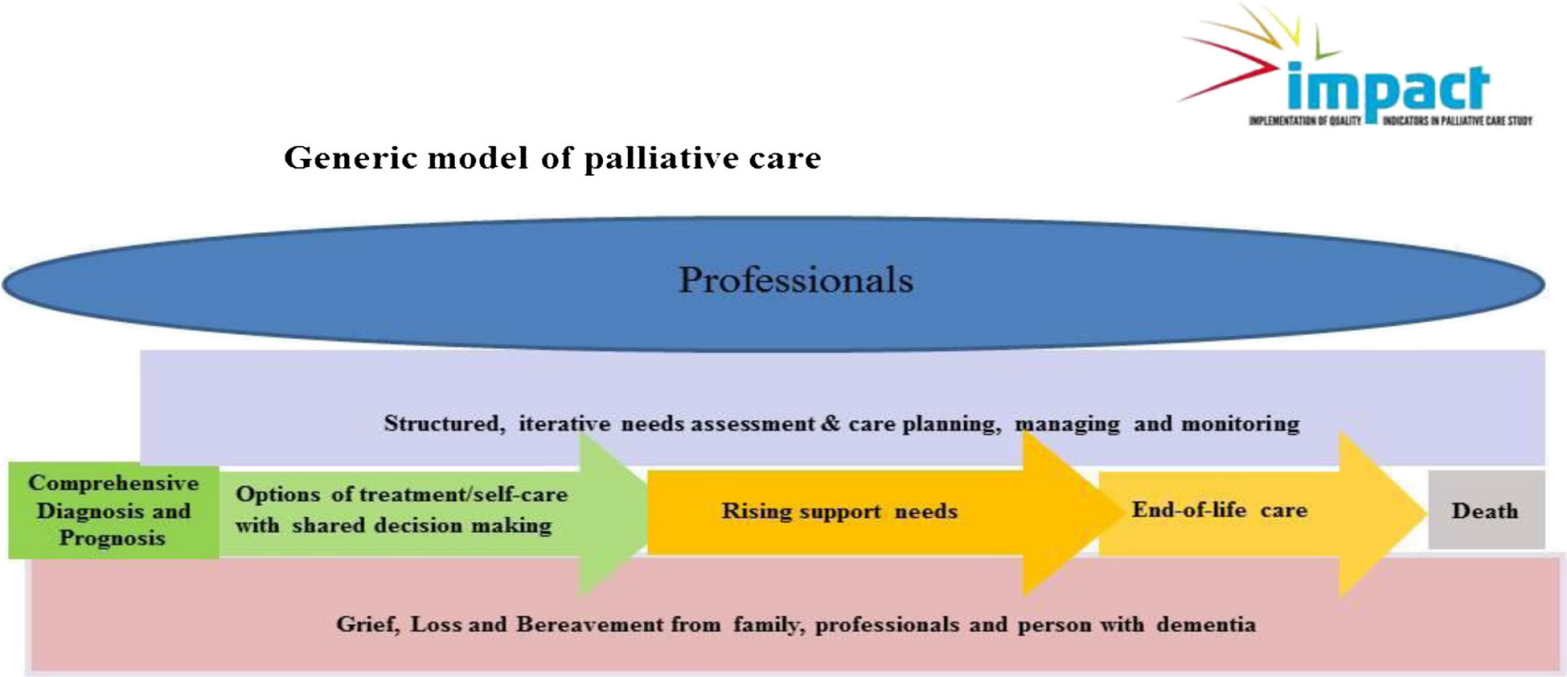
Generic model of palliative care.

The findings and outputs from Objective 4 are reported here. The basic generic model of palliative care is shown in Figure 2. The patient’s pathway moves from left to right, in the different coloured arrows. Each colour represents a different stage of the process of palliative care, but the transitions between stages may be less clear in practice.

The top layer of the model (in blue) represents the varied expertise brought to bear on palliative care. Specialists may be doctors, nurses, social workers, psychologists, occupational therapists, physiotherapists, speech therapists, pharmacists and other care staff from dedicated palliative care teams. Generalists may be family doctors, community nurses, family members and care workers. The professionals work within an iterative process of needs assessment and tailoring interventions, shown in lilac. The patient’s experience follows the arrows from diagnosis (in green) through progressive deterioration (in yellow) to death. Grief, loss and bereavement, experienced by all, is shown as the base layer of the model and can begin early in the disease process.

The landscape can be populated with quality indicators. For example, the top layer (Professionals) might include evidence of care coordination, community orientation or evidence of increased skill as markers of high quality professional practice. In the part of the patient’s pathway where support needs are rising, the use of the Gold Standard Framework, including systematic pain assessment, attention to psycho-social needs, nutrition assessment (including limited use of PEG/NG tubes), agreement on infection management and the deployment of prognostication tools might all function as quality indicators. Training and continuous learning, audits of outcomes and a stable leadership & workforce, with a rich staff skill mix, might suggest high quality care, as would the ascertainment of prior and current preferences, with carer or family involvement. Fidelity to prior and current preferences, symptom control, family satisfaction, the appropriateness of the setting (home or hospital), and psychosocial and spiritual needs being met could act as markers of “ a good death”. These examples are illustrative only and are not meant to be definitive quality indicators.

The synthesis process generated the following assumptions that underpin this model of palliative care and the quality indicators used to evaluate it. These are: 1) Most dementia diagnoses are made by specialists, but the model is not concerned with the diagnostic process, or the disclosure of diagnoses; 2) Diagnosis may lead to an ‘active treatment’ phase which may also be ‘late’ in the disease trajectory so that the patient enters palliative care directly; 3) Palliative care may begin before active treatment ceases; 4) Palliative care should be structured and managed pro-actively; 5) Bereavement, as experienced by family and support staff, may begin before death occurs.

This framework includes the active management of symptoms, attention to psycho-social needs and care co-ordination. Use of a structured end-of-life care process goes together with training and continuous learning among care providers, audit of outcomes (however defined) as a routine form of learning, a stable leadership and workforce amongst all providers, and sufficient staff skill mix amongst direct care providers. The model will be developed further in the next phase of the IMPACT project, in which it will be field tested to assess its value as a landscape within which a range of different quality indicators can be applied.

In developing the model the IMPACT research team identified a number of high-level aspects of the landscape which have an effect on quality of palliative care for people with dementia. These include the division of labour, coordination and responsibilities amongst practitioners of different disciplines and the implications of this for learning; the structure and function of care planning; the management of rising risk and increasing complexity; boundaries between disease-modifying treatment and palliative care; and the process of bereavement.

## Discussion

This model of palliative care for people with dementia and their carers creates a landscape in which quality indicators can be embedded systematically, in response to the changing nature of dementia as it progresses. It highlights a number of areas of practice where quality of care matters, including: the division of labour amongst practitioners of different disciplines and the implications of this for learning; the structure and function of care planning; the management of rising risk and increasing complexity; boundaries between disease-modifying treatment and palliative care and between palliative and end-of-life care; and the process of bereavement.

### Division of labour and its educational implications

The variable availability of specialists in palliative care means that much of the work of palliative and end-of-life care is carried out by generalists in medicine or nursing, or (in care homes and in people’s own homes) by care assistants whose training and access to support may be limited, and by family carers who may be well supported or may face the situation largely on their own. There are few published studies of educational programmes for improving palliative care for people with dementia, and where educational impact has been measured, no positive effects were demonstrated [38]. There was weak evidence [50] that those caring for patients with dementia felt they needed more training on the subject of palliative care, death and dying, and that learners felt that education changed their attitudes [51]. However, the highly diverse and often unclear educational interventions that have been evaluated have not shown any benefits for patients. In a review of education in palliative care for people with dementia, Mittman proposes training patients and carers to educate their physicians and other healthcare providers, a train-the-trainer approach for physicians to teach clinical skills to their colleagues, and a case study method for clinicians to present the principles of high quality dementia care [52]. The presence of educational programmes within palliative care services may be a useful quality indicator, especially in the care of patients with dementia, but caution is needed in thinking that any type of training will have positive outcomes. The law of inverse access to training also seems to apply; that is, that training is concentrated on those already in receipt of professional education. For example in England, among care staff, those working with people with dementia are the least trained of an under-trained workforce [28].

### Structure and function of care planning

Reviews of Advance Care Plans (ACP) suggest that their presence, acceptance, and their content can act as quality indicators for palliative care. Sampson et al [53] describe one early form of advance care planning in the United Kingdom (UK) and Australia, through examples of where a Power of Attorney (over financial matters) and ‘Let me Decide’ programme enabled the appointment of an attorney to make proxy decisions when the person with dementia no longer had capacity. In Australia a similar development led to a significant decrease in the transfer of nursing home residents to hospital with no changes in overall mortality [54]. However, people with dementia used to be less likely to have an Advance Care Plan (ACP) compared with those with cancer [55] In England, it will probably take some time for legal changes, such as the ability of a Lasting Power of Attorney to make health care decisions, if this has been agreed in advance, to be more acceptable among older people [56].

McCarthy and Addington-Hall [51] emphasise that identifying patients with future palliative care needs early should improve end-of-life care. Some argue that cognitive impairment should be assessed as early as possible, focusing on appreciation, reasoning and expression of consistent choices [57]. Not surprisingly, the reviews portray advanced care plans as more likely to be initiated and managed in centres with a specialised interest in palliative care but this is a rare care setting for people with dementia [58]. For instance, Shega et al’s review notes there are limited opportunities for people with dementia to use the services of hospices. They speculate that this may be because of a lack of awareness that dementia is a terminal illness and worries about not being able to manage behavioral symptoms [59,60]. Palliative care is often medicalised in hospital [61] possibly due to lack of advance decisions or directives meaning that inappropriate admissions or interventions may occur. Educating family carers of people with dementia about the clinical features and implications of advanced disease increases the likelihood that they will choose ‘comfort care’ for their relative rather than aggressive medical interventions [62].

### Managing risk and complexity

The progressive nature of dementia means that risks rise and the complexity of the conditions increases over time. These changes express themselves in decisions about pain control, nutrition, hydration, and (in dementia) psychological and behavioural symptoms, all of which might act as quality indicators of care.

The ‘gold standard’ for pain assessment is self-reporting but while many people with dementia can report pain effectively they may not be “heard” because of their diagnosis [63]. Because there is no evidence that pain among people with dementia produces any particular signs or behaviours that are unique [64], tools such as the Disability Distress Assessment Tool may be useful [65], alongside pain-specific tools like Doloplus2 [66]. The use of artificial nutrition for people with advanced dementia varies between countries and settings [67]. Sampson et al [50] note the lack of studies on the effect of these interventions on quality of life; however, they report evidence of increased morbidity and mortality from enteral feeding. Quality indicators relevant both to assessment tools and enteral feeding might therefore be useful.

Many people with dementia develop behavioural and psychological disturbances such as agitation, apathy, aggression, depression, delusions, wandering, sleep disturbances and hallucinations [68]. Aggressive behaviours increase, posing a concern for those providing end-of–life care [69]. Difficult behaviours, such as aggression and resistance to care, may also be indicators of unmet needs such as under-detected or under-treated pain, delirium or infection [50].

Factors that promote behavioural and psychological symptoms may be environmental (noise, temperature, space, light, presence of music and being restrained), biological and physical changes (lack of exercise, fatigue, hearing, vision impairment, pain, hunger, thirst and the frustration with being unable to meet one’s own basic needs) or psycho-social (lack of stimulation, reinforcement of negative behaviour, depression, fear, anxiety psychosis, and invasion of personal space) [70]. Roger’s 2006 review [67] concludes that better communication strategies and strong positive social relationships may decrease agitation and aggression and that caring and supportive environments may diminish aggressive outbursts by people with dementia.

### Boundaries

The interchangeability of concepts like palliative care, end-of-life care and terminal care has created confusion about their exact content, the stage of life to which they refer and the patients for whom they may be appropriate. Whilst palliative care is extensively described, the palliative care patient is not [71,72], and the description of this patient population remains vague [4]. In the World Health Organisation’s (WHO) definitions of palliative care, the patient is only defined as having a disease that is not responsive to curative treatment (WHO 1990) or a disease that is life-threatening (WHO 2002) [6]. Palliative care populations have therefore been defined in many different ways, in clinical practice, health policy and research [73]. The model presented here does not attempt to define patient populations, although we accept the need to do so. It does, however, assert that there are distinct and definable stages of disease-modifying treatment, palliative care and end-of-life care that have different content but that may also overlap.

### Bereavement and support

Bereavement may begin before death, during the period of palliative and end of life care [74,75]. Acknowledgement of this, and provision of support for relatives and staff, may be an indicator of quality. In addition to bereavement there are other areas of stress and distress about which we know very little. These may matter because staff may accumulate such experiences and because family carers are often likely to provide care to more than one member of their social network. Their professional experiences may also influence their personal roles and *vice versa*[76]. Little is known about the value of clinical supervision, peer support and reflection on practice for staff groups where supporting people with dementia at the end of life is increasingly becoming part of their routine work. There may be opportunities here for staff in dementia services to learn from palliative care practitioners, and for further quality indicators to be constructed around the wellbeing of practitioners.

## Summary

The co-design approach to developing a generic model of palliative care usable in different settings in different countries has been productive in several respects. It has considered the person needing palliative care as being on a journey within a landscape of services and professional disciplines. These services are much more than assemblies of doctors and nurses. They include those who give practical care, even if they do not see themselves as professionals; they also include the dying persons themselves, their friends, family and supporters, as experts.

The model as it currently stands represents activities that underpin quality in palliative care, like the division of labour, care planning, the management of risk and complexity, the salience of boundaries and the scale and scope of bereavement.

The model will be explored further in the next phase of the IMPACT project, to assess its suitability as a landscape within which quality indicators can be deployed.

## Competing interest

The authors declare that they have no competing interests.

## Authors’ contributions

SI, ND & JM lead the work-package on model development; MV-D & YE co-ordinated the IMPACT project; VRP, RS, EM, JB, LR, DH & LM contributed to data gathering and analysis at national sites; all authors contributed to constructing the models and drafting this report. All authors read and approved the final manuscript.

## Pre-publication history

The pre-publication history for this paper can be accessed here:

http://www.biomedcentral.com/1472-684X/12/30/prepub
